# Retroperitoneal Cecal Perforation Resulting from Obstructive Ascending Colon Adenocarcinoma

**DOI:** 10.1155/2020/9371071

**Published:** 2020-01-06

**Authors:** Daniel Paramythiotis, Anestis Karakatsanis, Moysis Moysidis, Diamantoula Pagkou, Petros Bangeas, Antonios Michalopoulos

**Affiliations:** ^1^1st Propaedeutic Surgery Department, AHEPA University General Hospital of Thessaloniki, Greece; ^2^1st University Surgery Department, Papageorgiou General Hospital of Thessaloniki, Greece

## Abstract

Most colorectal cancer patients in the early stages of the disease do not display any alarming symptoms. A total percentage of 9-27% of colorectal cancer patients present with acute abdomen, bowel obstruction, perforation, or bleeding. Perforation as the first presentation of the disease is seen in no more than 2.6-10% of patients. Intestinal perforation may be found on either the site of the tumor or on a more proximal site, caused by distention of the bowel due to peripheral obstruction. This is a case of a 75-year-old female patient who presents in the emergency department with retroperitoneal cecal perforation due to an obstructing tumor of the ascending colon. She underwent an emergency right hemicolectomy and washout of the retroperitoneal space. The cecum is not an unusual site of distention and subsequent perforation in the case of colonic obstruction, especially in the presence of a competent ileocecal valve. While the mechanism of diastatic cecal perforation is well described, it is the first time in the literature that this does not occur on the anterior surface of the organ. In our case, cecal perforation presents as a retroperitoneal abscess without peritoneal spillage. Nonetheless, it still carries a grim prognosis and urgent surgical intervention is needed.

## 1. Introduction

Colorectal cancer (CRC) is the most common gastrointestinal cancer, representing the 3^rd^ most common cause of death due to cancer among men and the 2^nd^ most common cause of death due to cancer among women [1]. Colorectal cancer, in early stages, progresses with only subtle symptoms or none at all. Only about 15–30% of CRC patients present with symptoms of acute abdomen such as perforation, obstruction, gastrointestinal bleeding, or formation of an abscess [2]. These complications are most common in older patients, typically in their 6^th^ and 7^th^ decade of life, while a worse prognosis is associated with their manifestation. The reported incidence of complete obstruction ranges from 8% to 40%, while being responsible for up to 85% of emergency colorectal operations, whereas the incidence of perforation ranges from 2.6% to 10% [3]. Intestinal perforation may occur either through the tumor site or in a proximal location as a complication of mechanical obstruction caused by the tumor (diastatic perforation). Diastatic perforation is defined as a blowout of the wall of the cecum caused by an overdistention which results from remote obstruction of the distal colon. These two types of perforation are more common in patients after radiotherapy or while receiving chemotherapy [4]. To the best of our knowledge, this is the first case report of a patient who presented with a retroperitoneal abscess caused by a diastatic cecal perforation, while the tumor itself was located in the ascending colon.

## 2. Case Report

**Figure 1:**
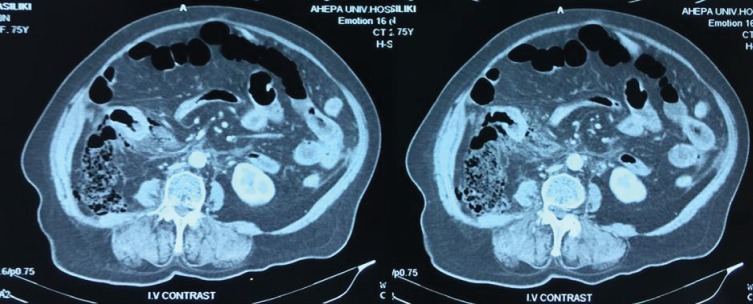
CT scan images showing retrocecal collection of fecal matter, without any presence of free air or fluid collection in the peritoneal cavity.

A 75-year-old female patient presented in the emergency department complaining of diffuse abdominal pain and difficulty in passing flatus for the last 48 hours. Clinical examination revealed abdominal distension and tenderness. On auscultation, there were no bowel sounds. Laboratory results showed mildly elevated white blood cell count (9.93 × 10^9^/L) and neutrophilia. An emergency CT scan of the abdomen was performed, which revealed free air and fluid collection in the retroperitoneal space in close relation to the cecum (Figure 1).

**Figure 2:**
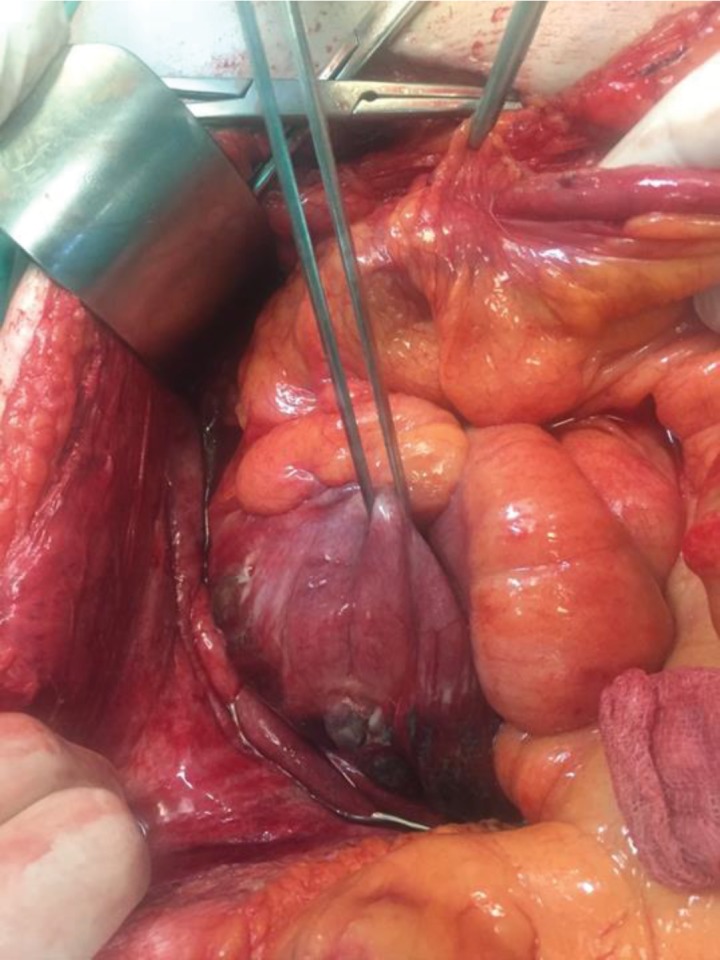
Exploratory laparotomy findings. Peritoneal cavity appears without spillage, but there can be seen a retroperitoneal collection.

**Figure 3:**
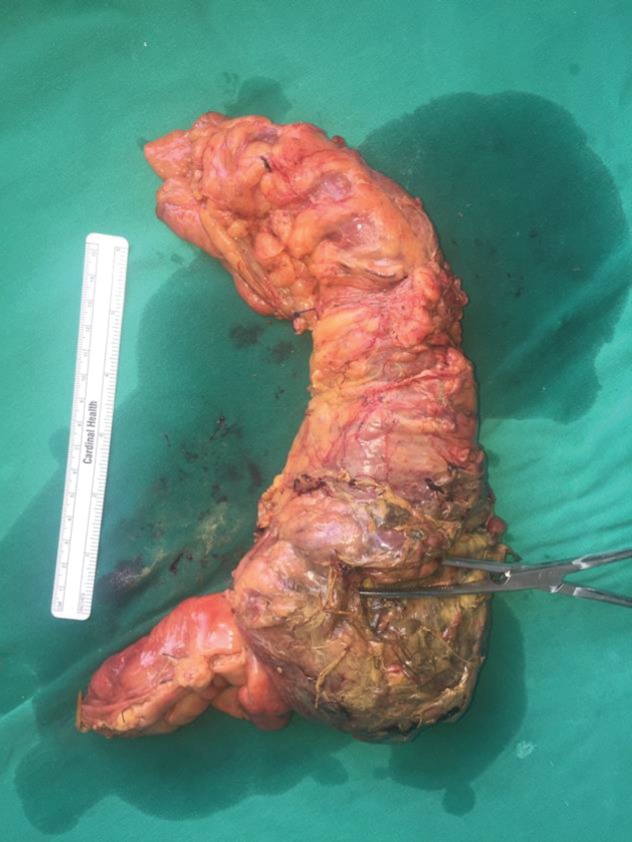
The right hemicolectomy specimen as it appears from the posterior side. A clamp is placed on the site of the perforation.

The patient underwent an emergency laparotomy, where an obstructing tumor was found in the ascending colon. No apparent spillage of the peritoneal cavity was to be found, but there was a contained perforation of the cecum in the retroperitoneum (Figure 2). A right hemicolectomy was carried out with primary side-to-side ileotransverse anastomosis, as well as a thorough washout of the retroperitoneal space (Figure 3). The patient was transferred to the surgical intensive care unit, where she passed away the following day due to septic shock. The final pathology report depicted adenocarcinoma of the ascending colon.

## 3. Discussion

Two types of perforation are associated with colon cancer: the direct perforation resulting from tumor necrosis and the perforation of proximal colon owing to distal obstruction caused by the tumor, which in the presence of a competent ileocecal valve, produces a closed-loop syndrome.

Direct perforations are the most common type, while diastatic perforations occur in a minority of CRC patients, as evident in the literature. Mandava et al. analyzed retrospectively 1551 CRC patients during a 10-year period and estimated that proximal perforation was present in 0.58% (9 out of 1551 patients) [5]. Carraro et al. reviewed a series of 83 consecutive CRC patients with perforation treated during a 14-year period at one institution and reported that 54 (65%) patients had a perforation of the tumor itself while 29 (35% of the perforations) had diastatic perforation proximal to an obstructing tumor [6]. Chen et al. published a 0.7% rate of proximal (≥2 cm cephalad to the tumor) perforation in a series of 1850 CRC patients (13 proximal perforations out of 1850 patients) [3], while Hennekinne-Mucci et al. reported that among 156 patients with acute left colonic obstruction, there were 27 cases with serosal tears of the cecal wall (17.3%) and 2 cases with apparent diastatic perforation (0.13%) [7]. Anwar et al., in a prospectively maintained CRC database of 762 consecutive patients, reported that 10 patients (1.31%) presented with acute colon perforation proximal to the tumor [8]. Lee et al. reported that among 1227 colorectal patients who were retrospectively analyzed, the rate of proximal perforation was 0.57% (7 patients) [9]. A larger percentage of diastatic perforation (6 out of 363 patients, 1.6%) was reported by Lu et al. in a retrospective review which tried to define the incidence of ileocolic ischemic changes due to the presence of an obstructive CRC [10]. Interestingly, Ozogul et al. reported that of a total of 223 patients who had colon cancer, 26 patients presented with a colonic perforation proximal to the tumor (11.66%), representing the largest reported proportion of patients to be admitted with diastatic colonic perforation [1]. In a study of Ngu et al., 10 out of 60 (16.7%) patients admitted with acute malignant left colon obstruction were reported with CT evidence of cecal wall pneumatosis, a sign of possible imminent perforation, while only one was recognized with perforation through the tumor site [11]. In conclusion, diastatic colonic perforation occurs in approximately 0.7-1% of CRC patients, regarding the study by Ozogul et al. as an outlier.

The cecum is the most common location of a diastatic perforation, owing to the fact that, according to the law of Laplace, “in a long pliable tube, the site of largest diameter requires the least pressure to distend.” Therefore, in the presence of distal large bowel obstruction, usually in the left colon, and with the prerequisite of a competent ileocecal valve, the cecum is the most common site of perforation [12]. In other words, the progression of bowel obstruction results in excessive dilatation of the cecum, which corresponds to the region of the colon with the maximum diameter and the greater wall tension. This dilatation may lead to bowel perforation, as excessive pressure is exercised on the vessels of its wall, resulting in hypoperfusion and secondary ischemia. Preexisting atheromatic disease and anemia may accelerate this process [10]. The subsequent perforation will cause fecal peritonitis and will accordingly increase the rates of morbidity and mortality in such cases. Cecal perforation will be found most often on the anterior longitudinal axis (unlike in our case), with sharp uninflamed margins [13]. Other common sites of diastatic perforation include the hepatic flexure, the splenic flexure, and the descending colon, while perforation of the transverse colon due to distal obstruction has only once been reported [12].

Risk factors heralding the progression of the ischemic changes to frank perforation include a cecal diameter of 12 cm, a long-standing dilation, and an intraluminal pressure greater than 80 mmHg. Additionally, a closed-loop bowel obstruction may only occur in the presence of a competent ileocecal valve, which barium studies have exhibited in only 10-30% of the tested population [13].

In case of complications arising from the presence of a CRC or even, as often is the case nowadays, in the evaluation of nonspecific abdominal pain (NSAP), computed tomography (CT) represents the most commonly utilized imaging technique that is also more readily available. In the case of an obstructing lesion, CT is a sensitive imaging modality and it may provide additional information on the transition point of the obstruction. In an obstructing colorectal cancer with a competent ileocecal valve (closed-loop obstruction), CT may identify the obstructing mass causing severe dilatation of the proximal colon, while the small bowel will not be dilated. In such cases, the finding of a cecal diameter over 12 cm or cecal pneumatosis is often interpreted as a sign of ischemia and imminent perforation of the cecum may ensue. Perforation of the colon may also be demonstrated by CT with the depiction of a defect in the colon wall that may be furthermore accompanied by a fluid-density abscess, free air, or stranding of the pericolic fat. Identifying the irregular wall thickening of the adjacent colon is critical for making the diagnosis of underlying colon cancer, thus differentiating a perforation caused by benign causes. However, in some cases, the severe pericolic inflammation accompanying a large abscess or peritoneal spillage may make it difficult to make an accurate preoperative diagnosis [14]. Another imaging characteristic that has been correlated to diastatic perforation of the colon is the presence of massive ascites (*V* > 2000 mL), probably due to diluted fecal material spilling through the perforation [15].

A retroperitoneal abscess may be of multifactorial origin, and it manifests usually secondary to infections of the gastrointestinal or genitourinary tract. If no definite etiology can be discovered, it is characterized as primary. Important predisposing factors are considered to be diabetes mellitus, muscle trauma, and individuals tested positive for HIV. The most common causes of retroperitoneal abscesses are infections of the duodenum, pancreas, terminal ileum, appendix, and ascending and descending colon. Retroperitoneal abscesses, in addition, may result from microbial agents such as tuberculosis, Staph. aureus, E. coli, Bacteroides species, or other rare bacteria, such as the Actinomyces species [16]. Since cecum (in the case of distal obstructing cancer) usually perforates most often on the anterior longitudinal axis [13], it is not surprising that there is only one more case report of a retroperitoneal diastatic cecal perforation, which manifested however as subcutaneous cervical emphysema and pneumomediastinum, and not as a retroperitoneal abscess [17].

The principles of damage control surgery may also apply when the surgeon confronts a diastatic colonic perforation, namely, source control and further resuscitation of the patient in the intensive care unit. A right hemicolectomy and an ileostomy with a distal colonic fistula without intraperitoneal anastomosis are usually warranted (in the case of a diastatic cecal perforation), as the anastomotic leak rate is very high in generalized peritonitis. In the frailest of the patients, the perforation may be exteriorized as a colostomy, providing thus source control and obviating the need for a major procedure [12]. In our case, drainage of the retroperitoneal space was also necessary for damage control. If the patient's situation permits, removal of the primary tumor following the principles of surgical oncology may be contemplated during the index surgery, provided however that there is no distant metastasis [17].

A diastatic perforation carries grim perioperative mortality, reported to be as high as 90%, as well as a worse long-term outcome, compared to tumor site perforation [2, 3, 12, 15]. Moreover, when a direct tumor perforation is encountered, the possibility of peritoneal cavity spillage with liquid feces is much less. This is partially due to compartmentalization of the spilled content from the peritumoral inflammation, as well as because the content of the distal colon is far less liquid compared to the right colon. Additionally, the patients with diastatic perforation always exhibit symptoms of intestinal obstruction preceding that catastrophe, such as starvation and dehydration, which may lead to renal insufficiency [15]. The perforation is, therefore, a “second hit” phenomenon that threatens to unbalance the already precarious condition of the patient.

## 4. Conclusion

Since the role of radiotherapy and chemotherapy in CRC is expanding, and due to the fact that the population is aging, the prevalence of diastatic colonic perforation may be on the rise. Although at present it is an infrequent event that many surgeons may not encounter during their whole careers, one should bear in mind that a pneumoperitoneum is not always due to tumor perforation and that an adequate search for the perforation site should include the whole colon.
